# Screening a thermotolerant *Komagataella phaffii* strain with methanol cross-tolerance and the associated physiological and transcriptional responses

**DOI:** 10.1016/j.synbio.2026.10.003

**Published:** 2026-10-15

**Authors:** Chaoyin Li, Tianzhi Jiang, Yesong Bo, Zhenghui Lu, Yuling Zhou, Guimin Zhang

**Affiliations:** a State Key Laboratory of Biocatalysis and Enzyme Engineering, College of Life Sciences, Hubei University, Wuhan, Hubei, 430062, China; b State Key Laboratory of Green Biomanufacturing, College of Life Science and Technology, Beijing University of Chemical Technology, Beijing, 100029, China

**Keywords:** *Komagataella phaffii*, Heat- and methanol-resistance, Protein expression, Transcriptomic analysis

## Abstract

*Komagataella phaffii* is a prominent methylotrophic yeast widely used in industrial biotechnology for heterologous protein production, chemical synthesis, and C1 feedstock utilization. However, its industrial application is limited by thermal stress and methanol toxicity, while the relationship between heat adaptation and methanol tolerance remains poorly understood. Here, we obtained a thermotolerant mutant ATH through ARTP mutagenesis followed by adaptive laboratory evolution (ALE) under heat stress (37 °C). ATH tolerated heat and methanol robustly under individual stress conditions, achieving 3-fold and 8-fold higher cell densities than GS115 at 37 °C or in 5% (v/v) methanol, respectively, yet failed under the combined stress of 37 °C and 5% methanol. Using three proteins (GFP, OPH, and ChiA) as reporters, we found that ATH showed higher heterologous expression than GS115 at 33 °C. Physiologically, ATH displayed enhanced cell wall/membrane integrity, reduced ROS accumulation and lower lipid peroxidation (>30% reduction in MDA), and accelerated formaldehyde clearance. Transcriptomics identified 561 shared differentially expressed genes, with coordinated upregulation of heat shock proteins (HSP70/40/90 families; Hsp104 and Hsp70-SSA2), antioxidant enzymes (catalase, peroxiredoxin), and cell wall/membrane remodeling genes, alongside downregulation of amino acid and secondary metabolite biosynthesis. Whole-genome resequencing further distinguished ATH from GS115 by only 19 variants (9 SNPs and 10 InDels), highlighting a compact genetic basis for the evolved phenotype. This work provides a *K. phaffii* chassis with improved individual heat and methanol tolerance and elucidates the physiological, transcriptional, and genomic mechanism underlying heat–methanol cross-adaptation.

## 1 Introduction

*Komagataella phaffii* (formerly known as *Pichia pastoris*) is widely used as a microbial cell factory for industrial production of heterologous proteins, owing to its high-density fermentation capacity, efficient post-translational modifications, and robust protein secretion. As a typical methylotrophic yeast, *K. phaffii* can utilize methanol not only as a sole carbon source but also as an inducer for the strong and tightly regulated alcohol oxidase 1 (AOX1) promoter, which is crucial for high-level recombinant protein expression [1]. Moreover, the use of methanol as a carbon source aligns with the national “dual carbon” strategy, as it enables the conversion of low-cost C_1_ feedstock into high-value products, contributing to the development of sustainable industrial biotechnology [2].

Despite its extensive industrial applications, *K. phaffii* still faces two critical bottlenecks that limit its economic feasibility and industrial efficiency. First, the optimal fermentation temperature of wild-type *K. phaffii* is around 28 °C, while massive heat is inevitably generated during high-density fermentation processes due to metabolic activities [3]. To maintain the fermentation temperature at the optimal level, industrial facilities have to rely on large-scale cooling systems, which consume enormous amounts of electrical energy and thus significantly increase the production costs [4]. Therefore, improving the thermotolerance of *K. phaffii* to allow fermentation at a slightly higher temperature (even a few degrees Celsius) would drastically reduce cooling energy consumption and lower industrial production costs. Second, although *K. phaffii* can metabolize methanol, its tolerance to high concentrations of methanol is relatively limited. Methanol is cytotoxic above the narrow concentration range (0.5%–2.5%) typically used for induction. Excessive methanol can also trigger reactive oxygen species (ROS) accumulation and oxidative stress, further inhibiting cell growth and protein expression [5,6]. Enhancing the methanol resistance of *K. phaffii* would enable the use of higher methanol concentrations in fermentation, which not only accelerates methanol metabolism but also maintains a higher specific growth rate under methanol-induced conditions, thereby improving industrial protein production efficiency and reducing production costs [5].

To address these challenges, several studies have focused on screening *K. phaffii* mutants with improved stress tolerance. For instance, Lin et al. isolated a *K. phaffii* strain G14 through mutagenesis combined with adaptive laboratory evolution (ALE) at 37 °C, which exhibited superior growth performance under heat stress compared to GS115. Notably, G14 was found to possess cross-tolerance to oxidative stress, as evidenced by reduced ROS levels and lipid peroxidation, along with enhanced peroxisomal catalase activity and enlarged peroxisome volume [7]. Subsequent work by the same group demonstrated that this oxidative-thermal cross-tolerant mutant also exhibited improved heterologous protein expression under methanol induction conditions, suggesting a link between stress tolerance and methanol-induced recombinant protein production [8]. On the other hand, Wang et al. screened a *K. phaffii* mutant strain MMC-SFC that tolerates 10% methanol through a combination of microbial microdroplet culture system (MMC) and shake flask culture (SFC) adaptive evolution, demonstrating efficient growth even under high methanol concentrations that are toxic to wild-type strains [5]. Nevertheless, these studies leave a significant gap: although G14 possesses oxidative-thermal cross-tolerance, its tolerance to methanol as a growth substrate was not systematically evaluated. Conversely, the methanol-tolerant strain MMC-SFC was not reported to withstand thermal stress. Consequently, whether heat adaptation confers cross-tolerance to high methanol concentrations in *K. phaffii,* and the underlying molecular mechanisms, remain poorly understood, limiting the rational engineering of robust industrial hosts.

Atmospheric and Room Temperature Plasma (ARTP) mutagenesis is an efficient and environmentally friendly physical mutagenesis method that can induce extensive genetic mutations in microbial genomes, thereby generating diverse mutant libraries with desirable phenotypes [9]; it has been successfully applied in the breeding of various industrial yeast strains, including *Phaffia rhodozyma* [10], *Candida tropicalis* [11], and *Saccharomyces cerevisiae* [12], to improve stress tolerance and product yield. Adaptive laboratory evolution (ALE), as a complementary strategy, can further stabilize and enhance the desired phenotypes of mutant strains by simulating long-term natural selection under specific stress conditions, allowing the strains to adapt to complex industrial fermentation environments through cumulative genetic changes [13].

In this study, we combined ARTP mutagenesis with ALE under heat stress to generate a thermotolerant *K. phaffii* mutant designated ATH. We evaluated its growth performance under individual and combined heat and methanol stresses, characterized the physiological changes associated with its enhanced tolerance, and analyzed the underlying molecular mechanisms through transcriptomic and genomic analyses. In addition, using three proteins as reporters, we assessed ATH as a host for heterologous protein expression at suboptimal temperatures. This study aims to advance understanding of the shared molecular architecture of heat–methanol cross-adaptation in methylotrophic yeasts and to provide a stress-resilient chassis for industrial biotechnology.

## 2 Materials and methods

### 2.1 Strains, plasmids, and culture media

The *K. phaffii* GS115 strain was used as the starting strain and preserved in our laboratory. The plasmid pHBM905M-*gfp* is also preserved in the laboratory. The culture media, including yeast extract peptone dextrose (YPD), buffered glycerol-complex (BMGY), buffered methanol-complex (BMMY), minimal dextrose (MD) and basal mineral salt media (BSM) were prepared following the protocols outlined in the Invitrogen Pichia Expression System manual (www.thermofisher.com). Luria-Bertani (LB) medium was used for bacterial transformation when propagating the plasmid.

### 2.2 ARTP mutagenesis and ALE

To obtain thermotolerant *K. phaffii* mutants, strain GS115 was cultured in 10 mL liquid YPD medium to the logarithmic growth phase, and the suspension was diluted to an OD_600_ of 1.0. Then, 10 μL of the suspension was spread onto a sterile iron plate and subjected to ARTP treatment (helium flow rate 10 L/min, RF power 120 W, treatment distance 2 mm) for 180 s to achieve approximately 90% lethality. The mutagenized cells were resuspended in 1 mL of physiological saline. An aliquot of the cell suspension was plated on YPD agar plates to count the number of colonies, while the remaining culture was incubated in YPD liquid medium and subjected to temperature-gradient screening (30 °C → 33 °C → 35 °C → 37 °C). Subsequently, further serial subculture was performed at 37 °C. The obtained culture was transferred to fresh YPD liquid medium with an initial OD_600_ of 0.1, incubated at 37 °C for 48 h, and then transferred to fresh medium under the same conditions. These steps were repeated approximately 20 times until high cell densities were obtained. The resulting cultures were spread onto YPD plates, and strains exhibiting robust growth were selected for subsequent analysis (Fig. 1A).

**Fig. 1:**
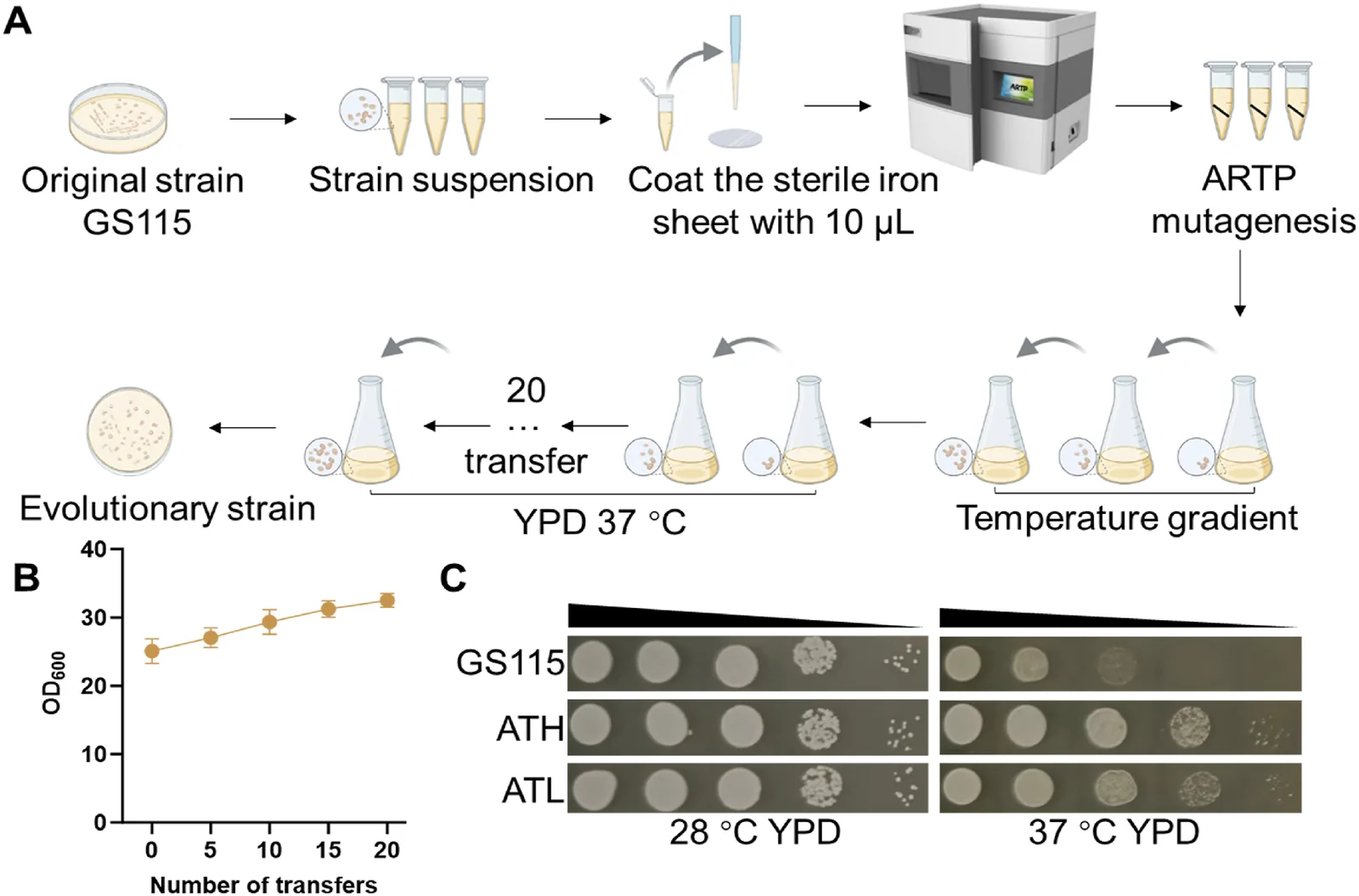
(**A**) Schematic diagram of the development of heat-tolerant *K. phaffii* strains using the integrated ARTP and ALE methods. (**B**) OD_600_ variation of the evolving population during adaptive evolution. (**C**) Heat-tolerance assay by a spot test. GS115, ATH and ATL strains were serially 10-fold diluted (10^0^ to 10^−4^), spotted onto YPD plates, and incubated at 28 °C (control) or 37 °C (heat stress) for 48 h. Diagram produced using BioRender.

### 2.3 Cell growth assay

#### 2.3.1 Temperature tolerance assay

Thermotolerance of the mutant strains and the parental strain GS115 was assessed using spot assays. Overnight cultures were harvested by centrifugation and resuspended in sterile water. Cell density was measured at 600 nm using a spectrophotometer. Samples with OD_600_ > 0.8 were appropriately diluted with fresh medium to ensure that measurements fell within the linear range (0.1–0.8), and the final OD_600_ values were calculated by multiplying the diluted readings by the dilution factor. The cell suspension was then adjusted to an OD_600_ of 1.0. After 10-fold serial dilutions, 5 μL of each dilution was spotted onto YPD plates and incubated at 28 °C or 37 °C. The mutant strain exhibiting the most vigorous growth at 37 °C was selected and named ATH. To further compare growth characteristics, ATH and GS115 were cultured in YPD, BMGY, and BMMY (with 1% v/v methanol) liquid media at 28 °C or 37 °C for 48 h. The initial OD_600_ of the cultures was normalized, and their growth curves were systematically assessed.

#### 2.3.2 Methanol tolerance assay

Strains ATH and GS115 were inoculated in BMMY medium supplemented with methanol at final concentrations of 1%, 5%, 6%, and 7% (v/v) with an initial OD_600_ of 1.0. The cultures were incubated at 28 °C or 37 °C for 48 h, and their growth curves were compared. To further evaluate methanol tolerance under sustained exposure, a semi-continuous methanol feeding experiment was subsequently performed. ATH and GS115 were cultured in BMMY medium under the same conditions, with the initial methanol concentration set at 1% or 5% (v/v) and methanol supplemented every 24 h to restore the respective initial concentration.

### 2.4 Evaluation of ATH as a heterologous expression host

To determine whether the enhanced stress tolerance of ATH translates into superior heterologous protein production, GFP, organophosphate hydrolase (OPH) and chitinase (ChiA) were expressed under the control of the methanol-inducible AOX1 promoter (P_AOX1_) in both strains. The corresponding expression cassettes were integrated into the *His4*^*-*^ locus of both strains via homologous recombination [14], generating the recombinant strains ATH-GFP/GS115-GFP, ATH-OPH/GS115-OPH, and ATH-ChiA/GS115-ChiA.

GFP was first used as a reporter to compare expression under different temperatures. ATH-GFP and GS115-GFP were inoculated into 50 mL of BMGY medium with an initial OD_600_ of 1.0 and cultured at 28 °C or 33 °C for 24 h. The cells were then harvested, resuspended in 25 mL of BMMY medium, and induced with 1% (v/v) methanol every 12 h. The fluorescence intensity of the fermentation broths was measured for three consecutive days using a microplate reader with an excitation wavelength of 485 nm and an emission wavelength of 525 nm, and the values were normalized to OD_600_ to account for differences in cell density.

OPH and ChiA were subsequently expressed in both strains under the same conditions at 33 °C to further assess the expression capability of ATH. OPH activity was measured using the methyl parathion assay [15], while ChiA activity was determined using the 3,5-dinitrosalicylic acid (DNS) method with colloidal chitin as the substrate [16].

### 2.5 High-cell-density cultivation of ATH

Bioreactor-scale high-cell-density cultivation of GFP-expressing strains was performed in a 5-L fermenter. ATH-GFP and GS115-GFP were first inoculated into a flask containing 200 mL of YPD medium and cultivated until the OD_600_ reached approximately 10. The cell cultures were then transferred into a 5-L fermenter containing 2 L of BSM medium for high-cell-density cultivation at 28 °C or 33 °C. Glycerol was supplied as the carbon source for cell growth, followed by methanol induction. During the cultivation, samples were collected and monitored every 12 h starting from methanol induction.

### 2.6 Physiological characterization of evolved strains

#### 2.6.1 Spot assay

The oxidative stress and cell wall integrity of the parental strain GS115 and the mutant strain ATH were assessed using spot assays. Overnight cultures of both strains were centrifuged, cells were collected and resuspended in sterile water, and the OD_600_ was adjusted to 1.0. Serial 10-fold dilutions were prepared, and 5 μL of each dilution was spotted onto YPD plates containing 8 mM H_2_O_2_, 50 μg/mL Congo Red (CR), or 50 μg/mL sodium dodecyl sulfate (SDS) [17]. All plates were incubated at 28 °C for observation.

#### 2.6.2 Propidium Iodide staining

To assess cell membrane integrity, *K. phaffii* strains GS115 and ATH were cultured in YPD medium to OD_600_ = 1.0. Cells were harvested, washed twice with phosphate-buffered saline (PBS, pH 7.4), and resuspended in the same buffer. 200 μL of the cell suspension was dispensed into black 96-well fluorescent microplates in triplicate. 5 μL of Propidium Iodide (PI, 5 mg/mL) was added to each well. The initial fluorescence intensity was recorded immediately, and the plate was subsequently incubated on a microplate shaker (150 rpm, 30 °C), with fluorescence measurements taken continuously for 1 h. PI fluorescence was detected using a microplate reader with excitation wavelengths of 535 nm and emission wavelengths of 615 nm. The fluorescence intensity was normalized to OD_600_ to obtain relative fluorescence units [18].

#### 2.6.3 ATPase activity assay

ATPase activity was determined using a commercial kit (A070-6-4, Nanjing Jiancheng Bioengineering Institute) according to the manufacturer's protocol [5]. Absorbance was measured at 636 nm using a spectrophotometer, and enzyme activity was expressed as the amount of inorganic phosphate (Pi) produced per milligram of protein per hour (μmol Pi/mg prot/h).

#### 2.6.4 ROS assay

Intracellular ROS levels were quantified using the fluorescent probe 2′,7′-dichlorodihydrofluorescein diacetate (DCFH-DA) [19]. Strains GS115 and ATH were cultured in BMMY medium supplemented with 1% or 5% (v/v) methanol and incubated at 28 °C or 37 °C, respectively. Samples were collected every 12 h, and cells were harvested by centrifugation, washed, and resuspended in PBS. Cell suspensions were incubated with 10 μM DCFH-DA dissolved in DMSO at 28 °C or 37 °C for 1 h in the dark. After dye loading, cells were collected, resuspended in PBS, and lysed using the glass bead disruption method. The supernatant was collected, and the fluorescence intensity of 2′,7′-dichlorofluorescein (DCF) was measured at an excitation wavelength of 480 nm and an emission wavelength of 525 nm. Fluorescence intensity was then calculated by normalizing the measured DCF fluorescence to the OD_600_ of each sample.

#### 2.6.5 MDA concentration

Malondialdehyde (MDA) content was determined using an MDA assay kit (Solarbio, China) [7]. After centrifugation, the cell pellets were resuspended in the extraction buffer and lysed using the glass bead disruption method. The supernatant was collected and assayed according to the manufacturer's protocol. Total protein concentration was determined using the Bradford method, and the MDA content was normalized to the protein concentration for comparative analysis.

#### 2.6.6 Formaldehyde concentration determination

Formaldehyde concentrations in the culture supernatants of strains GS115 and ATH were quantified using Nash reagent [20]. Both strains were cultured in BMMY medium containing 1% or 5% (v/v) methanol at 28 °C and 37 °C, respectively, with samples taken every 12 h. After centrifugation, 125 μL of the supernatant was transferred to a 96-well plate, mixed with 125 μL of Nash reagent (5 M NH_4_OAc and 50 mM acetylacetone), and kept on ice during loading. The microplate was then incubated at 37 °C for 1 h, and absorbance was measured at 412 nm. A standard curve was generated using formaldehyde with concentrations ranging from 0 to 1 mM prepared in the same culture medium, and the formaldehyde concentration in each sample was normalized to cell density and expressed as μM/OD_600_.

### 2.7 Transcriptome sequencing

Strains GS115 and ATH were cultured in BMMY medium supplemented with 1% or 5% (v/v) methanol at 28 °C or 37 °C for 24 h, followed by RNA sequencing analysis by Tsingke Biotechnology Co., Ltd. Differential expression analysis was conducted using DESeq2 with the Wald test. Genes with |log_2_FoldChange| ≥ 1 and false discovery rate (FDR) adjusted p-value < 0.05 (Benjamini-Hochberg method) were identified as differentially expressed genes (DEGs). Functional annotation of DEGs was carried out using the GO database, categorizing them into biological processes (BP), cellular components (CC), and molecular functions (MF). KEGG pathway enrichment analysis was performed with KOBAS. The sequencing libraries and their corresponding experimental conditions were as follows: ATH-28-1/GS115-28-1 (ATH/GS115, 28 °C, 1% methanol), ATH-28-5/GS115-28-5 (ATH/GS115, 28 °C, 5% methanol), and ATH-37-1/GS115-37-1 (ATH/GS115, 37 °C, 1% methanol).

### 2.8 Genome sequencing

Genomic resequencing was carried out on ATH and GS115 by Personal Biotechnology Co., Ltd. (Nanjing, China). Clean reads were aligned to the *K*. *phaffii* GS115 reference genome (RefSeq accession No. GCF_000027005.1) using BWA, and single-nucleotide polymorphisms (SNPs) and insertions/deletions (InDels) were identified using GATK, including local realignment and base quality-score recalibration.

### 2.9 Statistical analysis

All experiments were conducted in triplicate. Data are presented as mean ± standard deviation (SD) of n = 3 biological replicates. Statistical analyses were performed using GraphPad Prism software (Version 10.0). Depending on the experimental design, data were analyzed by two-way ANOVA followed by Bonferroni's multiple comparison test. Differences were considered statistically significant when *p* < 0.05.

## 3 Results

### 3.1 ARTP-ALE-driven screening of *K. phaffii* GS115 mutants with enhanced thermotolerance

To construct a thermotolerant strain, we developed an integrated strategy combining ARTP mutagenesis and ALE, using wild-type *K. phaffii* GS115 as the starting strain (Fig. 1A). Initially, GS115 cells were subjected to ARTP treatment. The mutagenized population was first recovered in YPD liquid medium at 30 °C and then subjected to temperature-gradient screening in YPD liquid medium to enrich thermotolerant variants. At this stage, the OD_600_ of the mutagenized population reached 25 after culture in YPD liquid medium at 37 °C for 48 h. Subsequently, this mixed population, which exhibited robust growth at 37 °C, was subjected to iterative adaptive evolution in YPD liquid medium at 37 °C for 20 consecutive passages (Fig. 1A). During the evolution process, cell growth was monitored every five passages. As shown in Fig. 1B, biomass gradually increased during evolution, with the final OD_600_ stabilizing at 31–33 by passage 20, compared with the initial OD_600_ of 25 at passage 0.

Subcultured cultures were serially diluted and spread on plates, which were then incubated at 37 °C to obtain single colonies. Two single colonies were selected and designated ATH and ATL. The cultures of the two strains were diluted to an OD_600_ of 1.0, followed by 10-fold serial dilution to 10^−4^. Serial dilutions (from 10^0^ to 10^−4^) were spotted onto YPD plates and incubated at 28 °C or 37 °C for 48 h. The results showed no difference in growth between GS115 and the two mutant strains at 28 °C. However, at 37 °C, the two mutant strains (ATH and ATL) still grew at the 10^−3^ and 10^−4^ dilutions, whereas GS115 showed no growth at these dilutions. Additionally, at 37 °C, the growth of ATH was significantly better than that of ATL at the 10^−2^ to 10^−4^ dilutions (Fig. 1C). Thus, ATH was selected as the representative thermotolerant host for subsequent experiments.

### 3.2 Evaluation of temperature and methanol tolerance of ATH strain

To assess growth under heat stress, ATH and GS115 were cultured in YPD, BMGY, and BMMY (1% v/v methanol) at 28 °C or 37 °C. At 28 °C, both strains showed comparable growth in YPD (OD_600_ ∼35 at 48 h) (Fig. 2A). However, ATH exhibited markedly enhanced growth kinetics in BMGY and BMMY, particularly in the methanol-containing BMMY medium, where ATH reached an OD_600_ exceeding 15 at 48 h compared with only 10 for GS115. At 37 °C, the advantage of ATH became even more pronounced (Fig. 2B). In YPD, GS115 suffered severe growth inhibition (OD_600_ < 20 at 36 h), whereas ATH maintained robust growth (OD_600_ > 30). In BMGY, ATH reached OD_600_ ∼30 at 36 h while GS115 only attained ∼10. Most notably, in BMMY at 37 °C, GS115 exhibited severe growth inhibition (OD_600_ < 5), whereas ATH continued to proliferate (OD_600_ > 10), indicating that ATH possesses superior growth adaptability and thermotolerance across diverse nutritional conditions, particularly under heat stress.

**Fig. 2:**
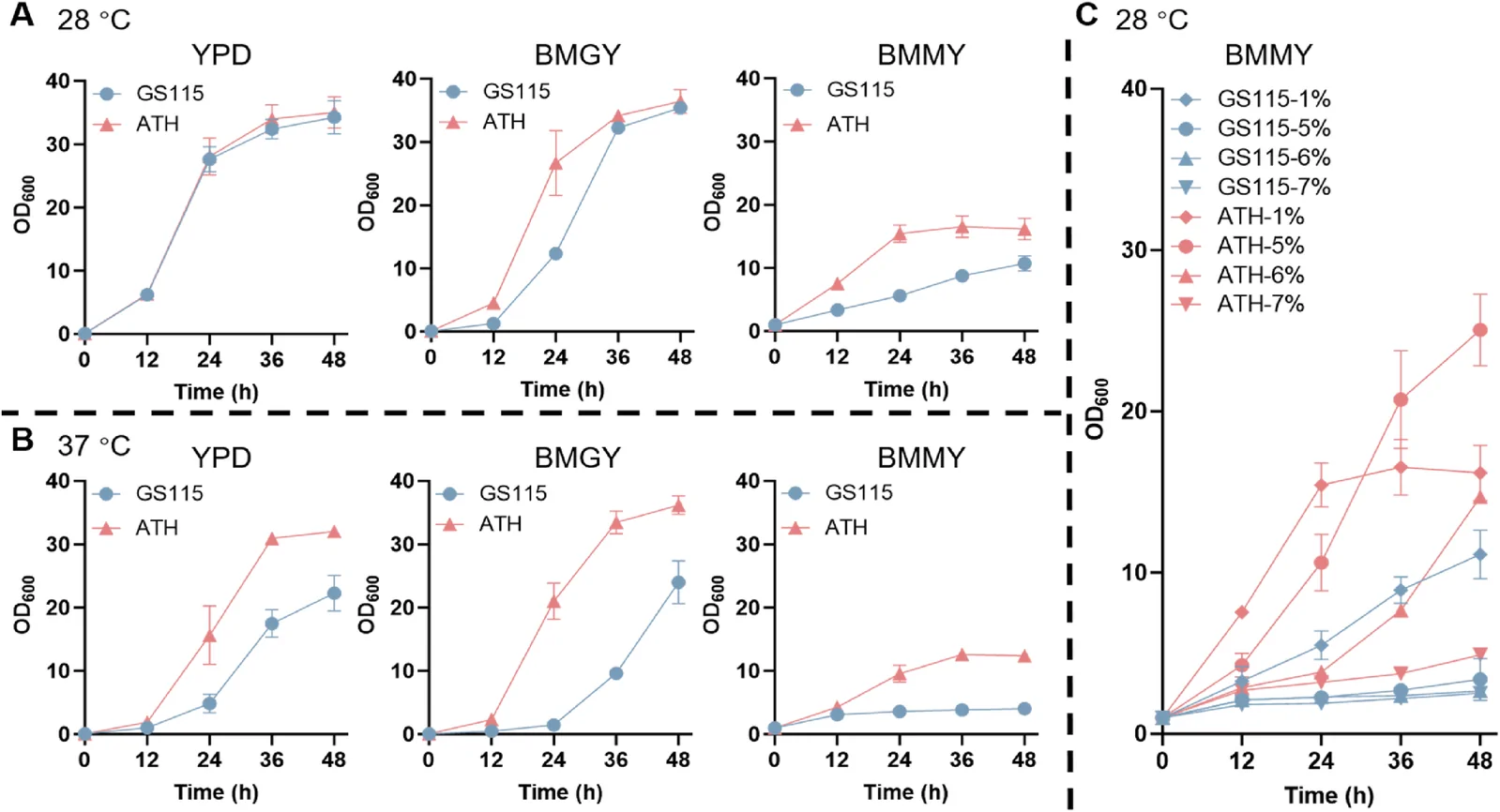
Growth curves of GS115 and ATH under various stress conditions. (**A**) Growth curves of strains cultured in YPD, BMGY, and BMMY media at 28 °C. (**B**) Growth curves of the strains cultured in YPD, BMGY, and BMMY media at 37 °C. (**C**) Growth curves of GS115 and ATH at 28 °C in BMMY medium supplemented with 1%, 5%, 6%, and 7% (v/v) methanol. Data are presented as mean ± SD (n = 3 biological replicates).

Encouraged by the robust growth of ATH in BMMY medium containing 1% (v/v) methanol, we hypothesized that heat adaptation might have concurrently enhanced methanol tolerance through shared stress-response pathways, and we therefore examined its growth under elevated methanol concentrations. ATH and GS115 were inoculated at an equal initial OD_600_ into BMMY medium containing 1%, 5%, 6%, or 7% (v/v) methanol and cultured at 28 °C for 48 h. As anticipated, ATH grew robustly at 5% methanol but showed inhibited growth at 6% and 7% (Fig. 2C), defining its upper methanol tolerance limit. Because methanol is volatile and continuously consumed by cells, the actual concentration may deviate substantially from the nominal value during cultivation. To rigorously evaluate methanol tolerance under sustained exposure, we performed a semi-continuous feeding experiment, in which methanol was supplemented to 1% or 5% (v/v) every 24 h to compensate for evaporative loss and metabolic consumption. Cell growth (OD_600_) was monitored periodically, with GS115 included as a parallel control. Under these conditions, ATH maintained a marked growth advantage over GS115 at both 1% and 5% methanol (Fig. S1).

We next evaluated the growth of both strains under combined stress. When subjected to the combination of 37 °C and 5% methanol, both ATH and GS115 exhibited severely inhibited growth, suggesting that simultaneous exposure to thermal and methanol stress surpasses the physiological limits of either strain (Fig. S2). Taken together, these results demonstrate that ATH possesses cross-tolerance to heat and methanol when each stress is applied individually, but not under the combined stress, and that its growth advantage over GS115 is maintained under sustained methanol exposure.

### 3.3 ATH as a heterologous expression host

To evaluate the industrial applicability of ATH as a protein expression host, single-copy expression cassettes for GFP, OPH, and ChiA under the control of the methanol-inducible P_AOX1_ promoter were introduced into both strains, and expression performance was compared.

Under standard induction conditions (28 °C, 1% methanol), ATH-GFP exhibited significantly higher GFP fluorescence intensity normalized to OD_600_ (19407 FLU/OD_600_) compared to GS115-GFP (15344.7 FLU/OD_600_) at 72 h post-induction, representing a 26.5% increase (*p* < 0.0001, Fig. 3A). Given ATH's robust growth at 37 °C, we initially assessed GFP expression at this temperature. Although ATH-GFP still exhibited significantly higher fluorescence intensity (4110.2 FLU/OD_600_) than GS115-GFP (2859.1 FLU/OD_600_), representing a 43.8% increase (*p* < 0.0001, Fig. S3), both strains showed markedly reduced fluorescence relative to 28 °C, suggesting that protein synthesis or folding capacity was severely impaired at this temperature. Therefore, we selected 33 °C as a suboptimal expression temperature, representing a compromise between enhanced growth and functional protein synthesis. At 33 °C with 1% methanol induction, ATH-GFP achieved a specific fluorescence intensity of 16039.6 FLU/OD_600_ at 72 h, significantly higher than that of GS115-GFP (11282.5 FLU/OD_600_) (*p* < 0.0001), representing a 42.2% improvement (Fig. 3B). Notably, the expression level of ATH-GFP at 33 °C remained comparable to that of GS115-GFP at the standard 28 °C.

**Fig. 3:**
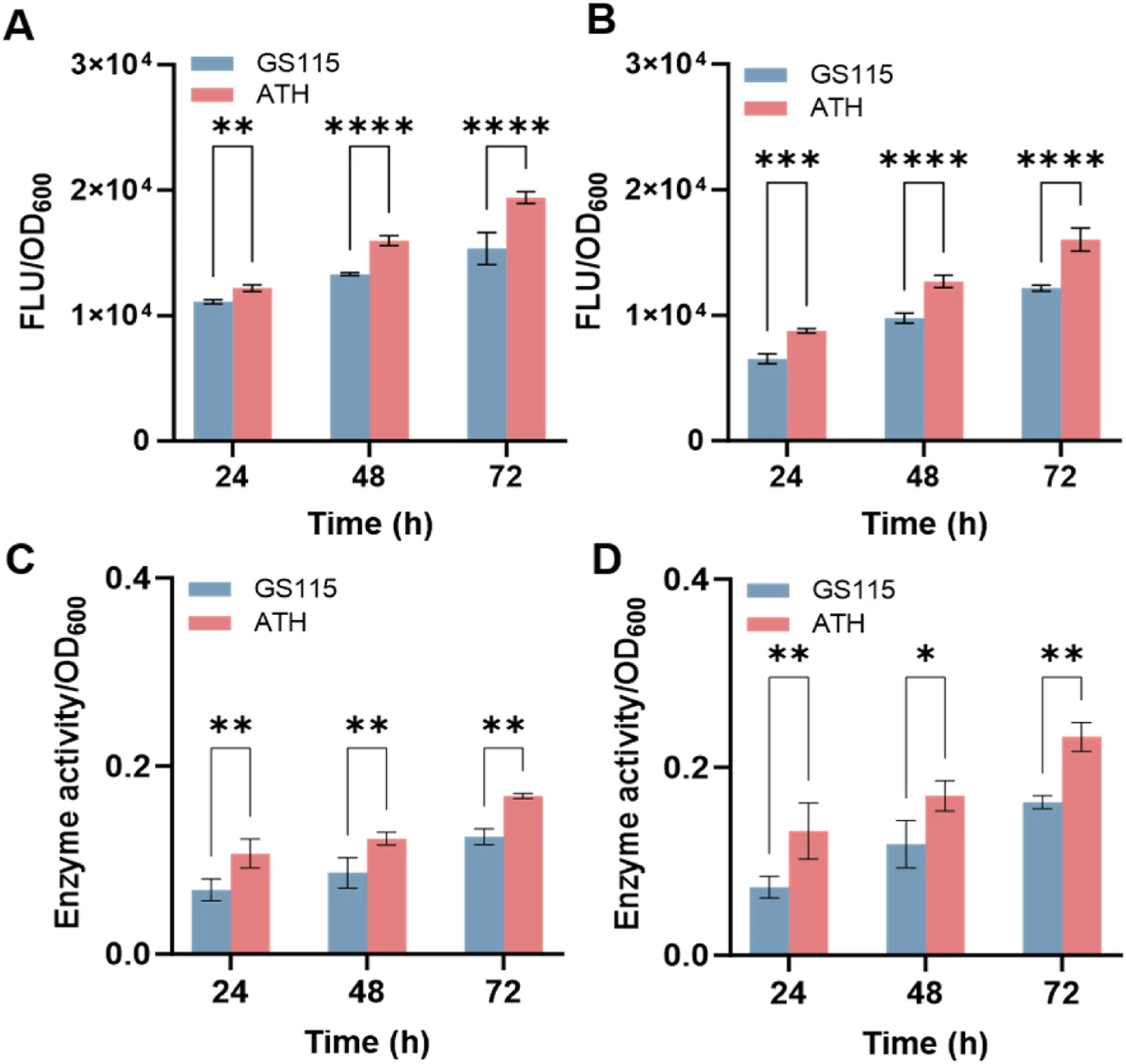
Protein expression in GS115 and ATH strains. GS115-GFP and ATH-GFP were fermented in BMMY medium for 72 h, with 1% (v/v) methanol added every 12 h, and cultured at 28 °C (**A**) and 33 °C (**B**), respectively. (**C**) OPH activity in the fermentation supernatant during induction. (**D**) ChiA activity in the fermentation supernatant during induction. Data are presented as mean ± SD (n = 3 biological replicates). ****P < 0.0001, ***P < 0.001, **P < 0.01, *P < 0.05.

We then expressed OPH and ChiA at the same 33 °C induction temperature to determine whether ATH's advantage extends beyond a fluorescent reporter to functionally diverse heterologous proteins. For both enzymes, the ATH-derived strains exhibited markedly higher enzyme activities than their GS115 counterparts (Fig. 3C and D), demonstrating that ATH's superior expression capacity is not reporter-specific.

### 3.4 High-cell-density cultivation of ATH

ATH-GFP and GS115-GFP were further evaluated for high-cell-density cultivation in a 5 L fermenter. During the glycerol feeding phase, cell density was monitored, and feeding was terminated once the OD_600_ reached 100. After glycerol feeding was stopped, the methanol induction phase was initiated and lasted for 120 h. The GFP fluorescence of ATH-GFP and GS115-GFP was compared at different temperatures. At 28 °C, ATH-GFP exhibited an approximately 21% increase over GS115-GFP after 120 h of fermentation (Fig. 4A). Although GFP expression in both strains decreased at 33 °C, ATH-GFP still maintained a significantly higher fluorescence level, showing an approximately 30.5% increase after 120 h (Fig. 4B). These results indicate that ATH can improve heterologous protein expression levels and enable protein induction at elevated temperatures.

**Fig. 4:**
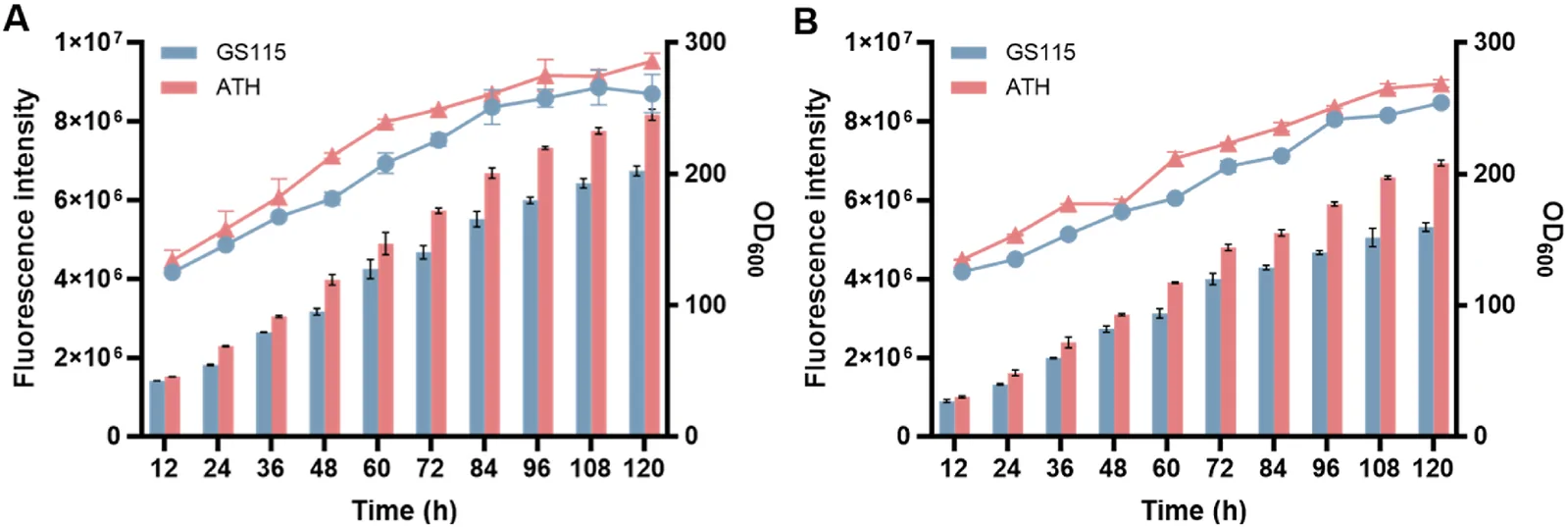
High-cell-density cultivation of GS115 and ATH strains. Growth curves (OD_600_) and GFP fluorescence intensity of fermentation supernatant at different induction time, cultured at 28 °C (**A**) and 33 °C (**B**), respectively. Data are presented as mean ± SD (n = 3 biological replicates).

### 3.5 Physiological characterization of the ATH mutant

To elucidate the mechanistic basis for the enhanced methanol and temperature tolerance of the ATH mutant, we assessed three key aspects of cellular stress defense: cell wall/membrane integrity, antioxidant capacity, and formaldehyde metabolism. The cell envelope provides the first line of defense, while ROS and formaldehyde are inevitable byproducts of methanol metabolism that must be efficiently managed to prevent cellular damage. The following assays were performed to compare ATH and GS115 across these parameters.

#### 3.5.1 Assessment of cell wall and membrane integrity in the ATH strain

To evaluate whether the enhanced stress tolerance of ATH was associated with improved cell wall and membrane integrity, we examined its sensitivity to two chemical stressors: CR and SDS. CR specifically interferes with cell wall polysaccharide assembly, while SDS disrupts the plasma membrane lipid bilayer [18]. CR and SDS were added to YPD medium plates at a concentration of 50 μg/mL. As shown in Fig. 5A, after 48 h of incubation, the ATH strain maintained good growth under both CR and SDS stress, whereas GS115 showed significant growth inhibition. These results indicate that enhanced cell wall and membrane integrity substantially contribute to the stress tolerance of the ATH mutant.

**Fig. 5:**
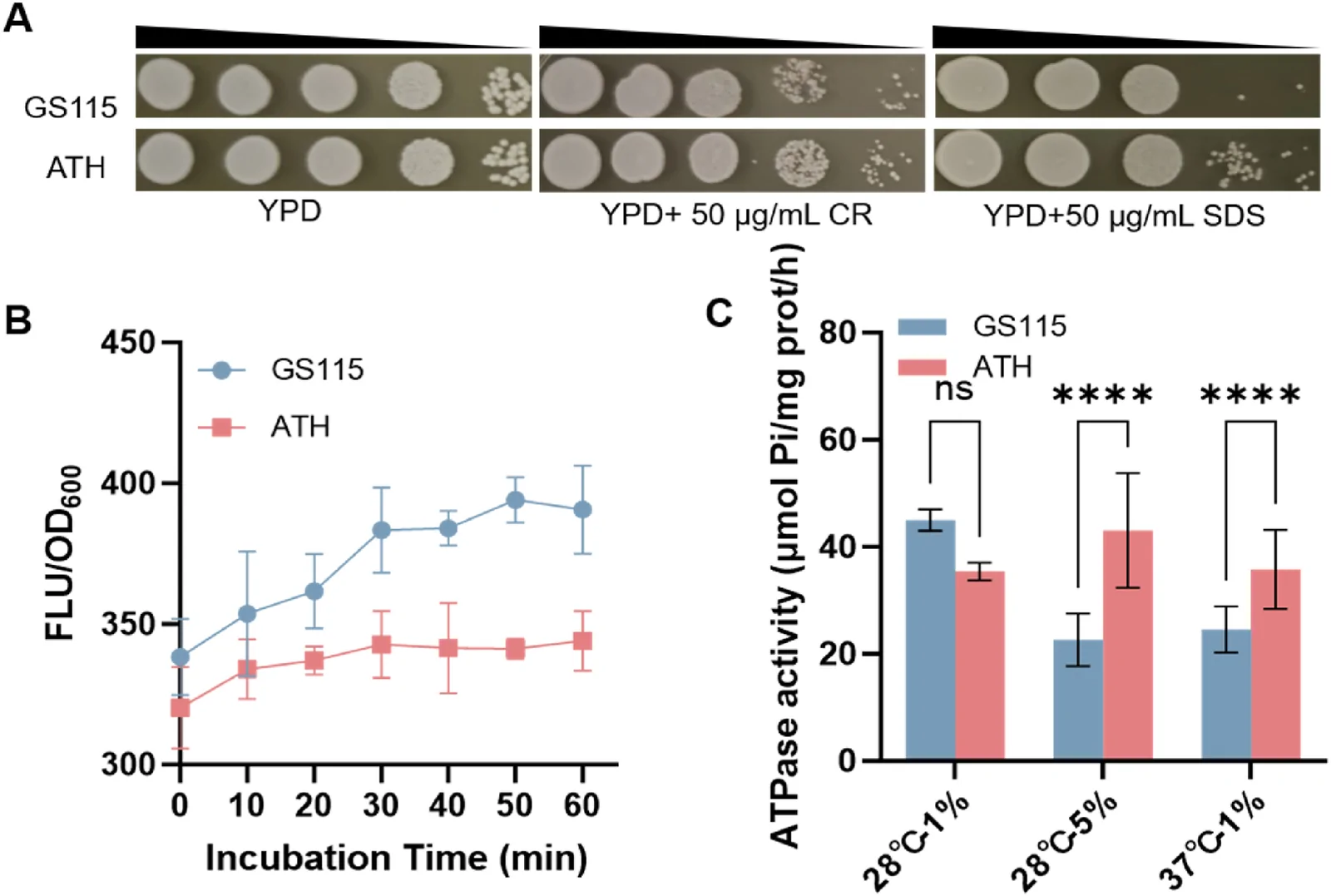
Multi-parameter assessment of cell wall and cell membrane integrity of ATH strain. (**A**) Sensitivities of GS115 and ATH to CR (50 μg/mL) and SDS (50 μg/mL) were evaluated by spot assay using 10-fold serial dilutions (10^0^ to 10^−4^) after 48 h of culture at 28 °C. (**B**) Membrane permeability of GS115 and ATH assessed by PI fluorescence. (**C**) ATPase activity on the membrane after culturing for 24 h under three culture conditions (28 °C with 1% methanol, 28 °C with 5% methanol, and 37 °C with 1% methanol). Data are presented as mean ± SD (n = 3 biological replicates). ****P < 0.0001, ***P < 0.001, **P < 0.01, *P < 0.05.

PI fluorescence staining was used to evaluate membrane integrity because it can penetrate only damaged membranes and intercalates into nucleic acids, yielding quantifiable fluorescence that reflects the degree of membrane damage [21]. As shown in Fig. 5B, during the 1 h monitoring period, the PI fluorescence intensity of GS115 was significantly higher than that of ATH, indicating that ATH possesses superior membrane integrity and stability. Membrane function was further assessed by measuring membrane-bound ATPase activity, a biochemical marker of both membrane integrity and cellular energetic status. Elevated ATPase activity reflects better membrane integrity and more active energy metabolism, enabling cells to maintain ion homeostasis and metabolic function under stress [5]. Under standard conditions (28 °C, 1% methanol), the ATPase activities of the two strains were comparable. However, under 5% methanol stress, the ATPase activity of ATH was 89% higher than that of GS115 (43.06 vs. 22.62 μmol Pi/mg prot/h), and at 37 °C, the ATPase activity of ATH was also 46% higher than that of GS115 (35.78 vs. 24.57 μmol Pi/mg prot/h) (*P* < 0.0001, Fig. 5C). Together, the PI staining and ATPase activity assays consistently indicated that ATH possesses stronger membrane structural integrity, which helps maintain cellular structural and functional stability under stress and thus contributes to its thermotolerance and methanol resistance.

#### 3.5.2 Oxidative stress tolerance

Methanol oxidation and heat stress inevitably generate ROS and formaldehyde, which synergistically damage membrane lipids, proteins, and DNA [22,23]. To dissect the antioxidant capacity of ATH, we first assessed its tolerance to exogenous H_2_O_2_. GS115 and ATH were spotted at ten-fold serial dilutions (10^0^ to 10^−4^) onto YPD plates containing 8 mM H_2_O_2_ and incubated for 48 h. As shown in Fig. 6A, ATH exhibited markedly enhanced survival compared with GS115, indicating a robust overall oxidative stress defense.

**Fig. 6:**
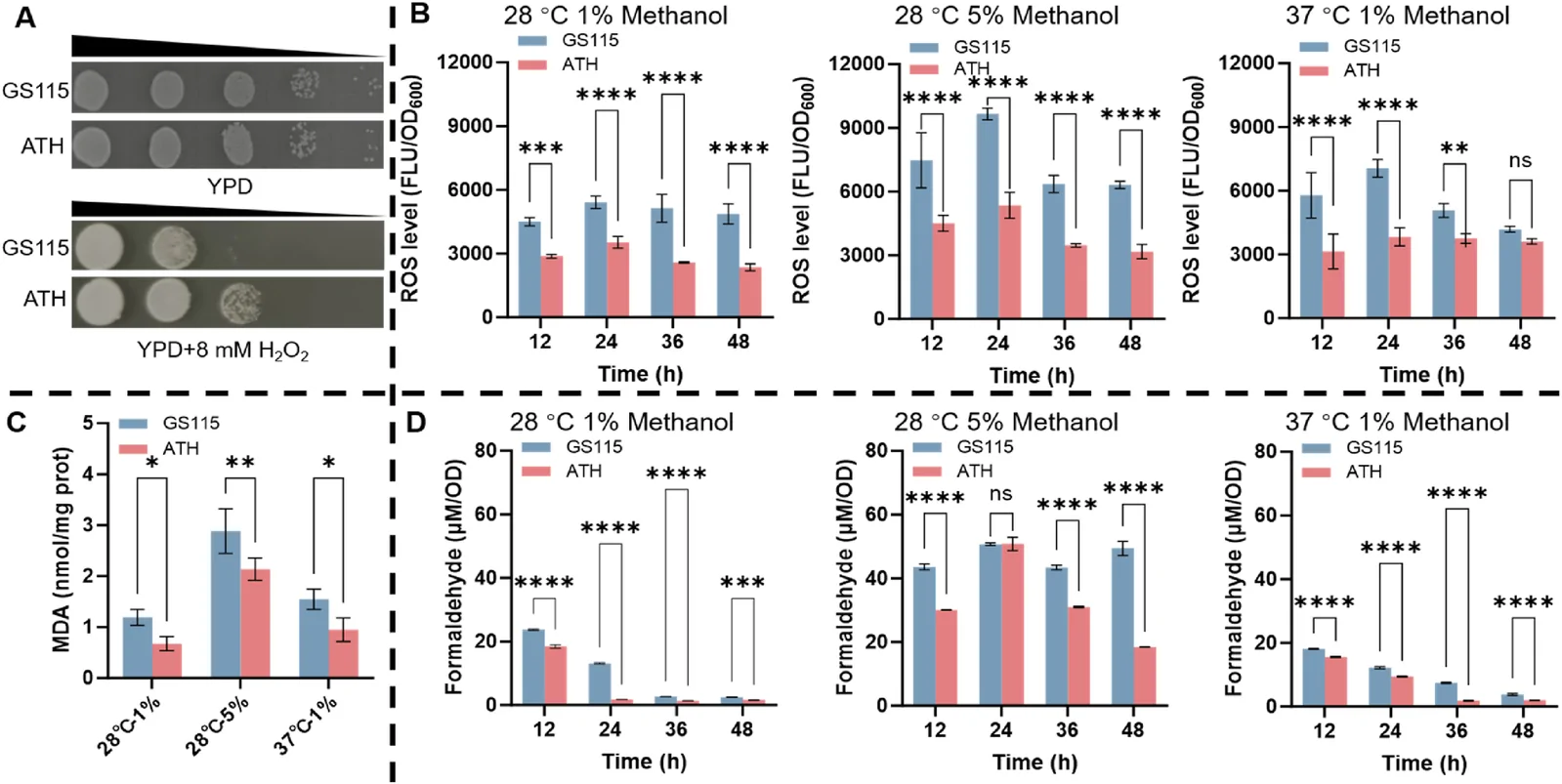
Evaluation of oxidative stress response in GS115 and ATH strains. (**A**) Growth of GS115 and ATH with the addition of 8 mM H_2_O_2_. (**B**) Measurement of ROS accumulation (FLU/OD_600_) in GS115 and ATH during fermentation at 28 °C-1% methanol, 28 °C-5% methanol, and 37 °C-1% methanol, respectively. (**C**) Intracellular MDA content of GS115 and ATH after 24 h of culture at the different culture conditions. (**D**) Extracellular formaldehyde level (μM/OD_600_) of GS115 and ATH at the different culture conditions. Data are presented as mean ± SD (n = 3 biological replicates). ****P < 0.0001, ***P < 0.001, **P < 0.01, *P < 0.05.

We next assessed whether the enhanced tolerance was accompanied by reduced intracellular ROS burden. Intracellular ROS levels were measured under three culture conditions: 28 °C with 1% methanol, 28 °C with 5% methanol, and 37 °C with 1% methanol. The results showed that ROS levels in GS115 gradually increased with rising temperature and methanol concentration, whereas ROS accumulation in ATH was significantly lower than in GS115, decreasing by approximately 50% after 24 h under all stress conditions (Fig. 6B). Consistent with reduced ROS burden, lipid peroxidation, as quantified by MDA content, was also significantly attenuated in ATH. Under the same stress conditions (5% methanol or 37 °C), the MDA level in ATH was more than 30% lower than that in GS115 (Fig. 6C), indicating that membrane lipid peroxidation was substantially alleviated.

Since formaldehyde is both a direct cytotoxin and a potent inducer of ROS, we further compared formaldehyde metabolic clearance between the two strains. Extracellular formaldehyde was monitored at different temperatures and methanol concentrations over time. Under standard conditions (28 °C, 1% methanol), ATH maintained lower formaldehyde levels with faster clearance. Under 5% methanol stress, despite a similar peak at 24 h, ATH rapidly cleared formaldehyde thereafter. Under heat stress (37 °C, 1% methanol), ATH also exhibited faster formaldehyde clearance than GS115, particularly at 36–48 h (Fig. 6D).

### 3.6 Transcriptome analysis reveals the molecular basis of heat-tolerance and high methanol tolerance in the ATH strain

To identify the molecular basis of enhanced stress tolerance in ATH, we performed RNA-seq comparing ATH and GS115 under three conditions: 28 °C with 1% methanol (GS115-28-1 vs. ATH-28-1), 28 °C with 5% methanol (GS115-28-5 vs. ATH-28-5), and 37 °C with 1% methanol (GS115-37-1 vs. ATH-37-1), with three biological replicates per condition. The Q20 and Q30 values of clean reads exceeded 97% and 93%, respectively (Table S1), confirming data reliability. DEGs were defined as those with |log_2_FC| ≥ 1 and padj < 0.05.

Comparisons under heat stress (GS115-37-1 vs. ATH-37-1) and methanol stress (GS115-28-5 vs. ATH-28-5) identified 924 and 1555 DEGs, respectively. Venn diagram analysis revealed that 561 DEGs were shared between the two stress conditions (Fig. 7A), suggesting that ATH employs overlapping transcriptional responses to cope with thermal and methanol stress.

**Fig. 7:**
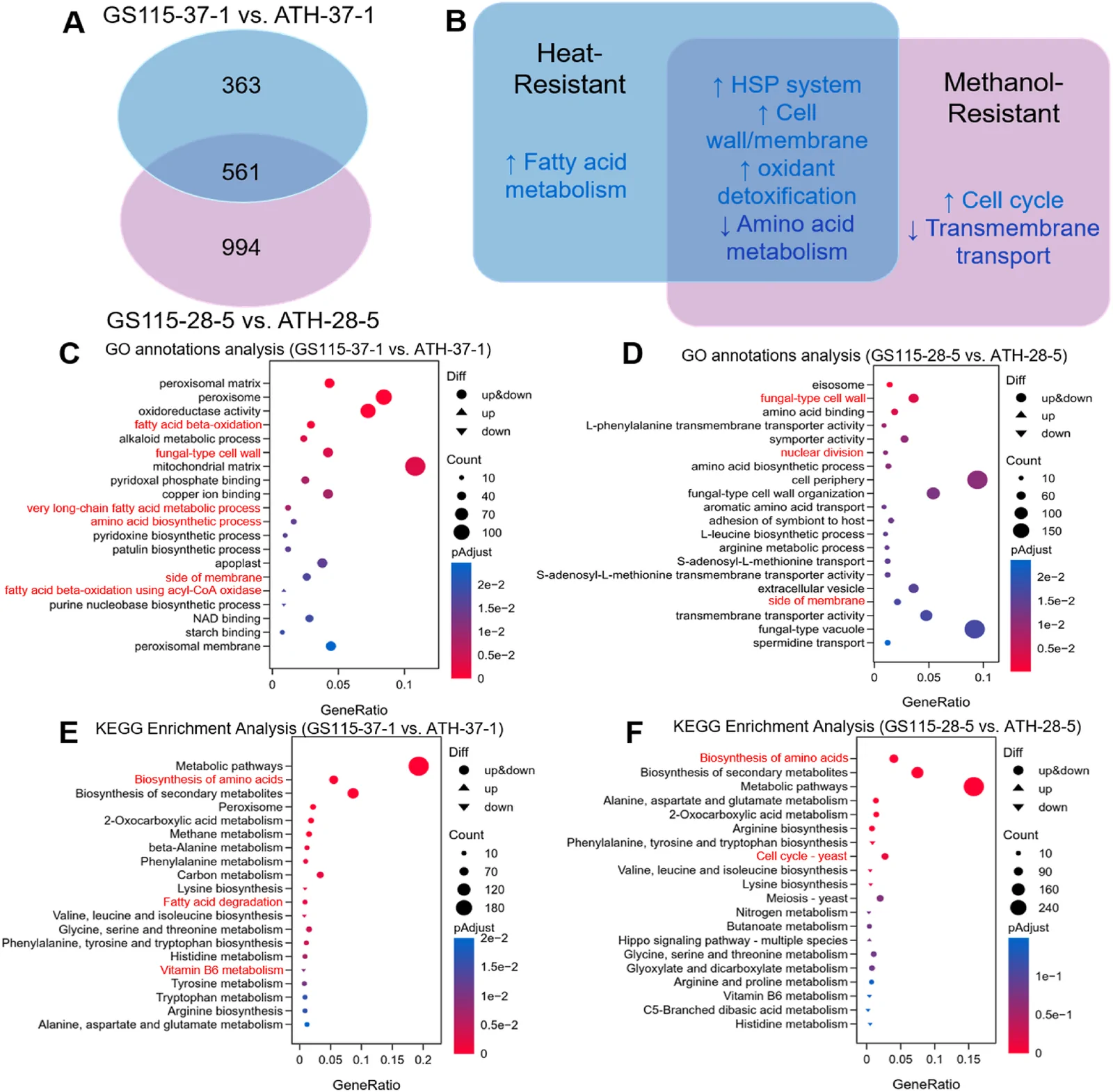
Transcriptomic analysis of GS115 and ATH strains. Functional enrichment analysis of DEGs: (**A**) Venn diagram of DEGs among GS115-37-1 vs ATH-37-1 and GS115-28-5 vs ATH-28-5 comparison groups. (**B**) Schematic diagrams of common and respective regulatory mechanisms underlying thermotolerance and methanol tolerance. (**C**) GO terms for GS115-37-1 vs. ATH-37-1 comparison, (**D**) GO terms for GS115-28-5 vs. ATH-28-5 comparison, (**E**) KEGG pathways for GS115-37-1 vs. ATH-37-1 comparison, (**F**) KEGG pathways for GS115-28-5 vs. ATH-28-5 comparison.

#### 3.6.1 Common transcriptional responses to heat and methanol stress

To dissect shared adaptive mechanisms underlying heat and methanol stress in ATH, we performed GO and KEGG enrichment analyses on the 561 shared DEGs. These genes were predominantly enriched in functional categories related to protein folding and quality control, cell wall/membrane organization, oxidant detoxification, and lipid metabolism (Fig. 7B), indicating a systems-level reprogramming of cellular defense networks. Notably, 11 heat shock protein (HSP) genes were significantly upregulated in ATH under both stress conditions, including members of the HSP70, HSP40, and HSP90 families (Table S2). Among these, PAS_chr1-3_0102 (Hsp104), PAS_chr4_0552 (Hsp70-SSA2) and PAS_chr2-2_0151 (SIS1) showed over 3-fold upregulation, indicating enhanced protein quality control under stress.

Beyond protein homeostasis, GO enrichment revealed significant upregulation of fungal-type cell wall (GO:0009277) and side of membrane (GO:0098552) categories (Table S3). Within the cell wall category, genes involved in β-1,3-glucan remodeling, including two β-1,3-glucanosyltransferases (PAS_chr1-3_0226 and PAS_chr1-3_0227), a major exo-1,3-β-glucanase (PAS_chr2-1_0454), a putative chitin transglycosidase (PAS_chr4_0559), and the mannoprotein PIR3 (PAS_chr2-1_0887), were markedly upregulated. Additionally, the O-glycosylated protein required for cell wall stability (PAS_chr4_0305) showed the highest fold-changes in both comparison groups. Other upregulated genes included the GPI-anchored cell wall protein PAS_chr2-2_0148, the seripauperin/TIP1 family protein PAS_chr4_0972, the glycosyl hydrolase PAS_chr1-3_0229, and the WD repeat-containing protein PAS_chr2-1_0355. Membrane-associated genes were also significantly enriched. Upregulated genes included the dicarboxylic amino acid permease PAS_chr4_0287, the subtilase family protein PAS_chr2-1_0172, the cysteine-rich secretory protein PAS_chr3_0076, and the hemoglobin-binding protein PAS_chr4_0151. Conversely, the mucin-like protein PAS_FragB_0067, the histidine phosphatase PAS_chr4_0493, and the flocculation protein FLO1 (PAS_chr3_0015) were downregulated. Collectively, these changes point to extensive remodeling of the cell envelope in ATH under both thermal and methanol stresses.

Concurrently, cellular oxidant detoxification (GO:0098869) was significantly enriched. Among the DEGs, catalase (PAS_chr2-2_0131), protein adenylyltransferase SelO (PAS_chr1-3_0160), and mitochondrial peroxiredoxin (PAS_chr3_0906) were upregulated, whereas carboxymuconolactone decarboxylase family protein (PAS_chr3_0355), omega class glutathione transferase (PAS_chr2-1_0315), and thiol peroxidase (PAS_chr2-2_0382) were downregulated (Table S4). Additionally, formaldehyde clearance-related genes showed differential expression between the two strains. Under heat stress, AOX, FLD, FGH, FDH, DAS, and CAT were upregulated in ATH, whereas GSH1/GSH2 were downregulated. Under methanol stress, AOX and CAT were upregulated, while GSH1/GSH2 were also upregulated (Table S5).

In contrast, amino acid biosynthesis (ko01230) and secondary metabolite synthesis (ko01110) pathways were significantly downregulated in ATH under both stresses (Fig. 7E and F, Tables S8–9), which may suggest a metabolic shift away from energy-intensive anabolic processes toward stress defense [24].

#### 3.6.2 Heat-specific transcriptional changes

Among the 363 DEGs specific to the heat-stress comparison (GS115-37-1 vs. ATH-37-1), multiple functional categories were enriched, including peroxisome organization, oxidoreductase activity, and amino acid metabolism (Fig. 7C–E). Fatty acid metabolism-related pathways were also significantly upregulated, encompassing fatty acid degradation (ko00071), β-oxidation (GO:0006635, GO:0033540), and very long-chain fatty acid metabolism (GO:0000038) (Tables S6 and 8). This aligns with reports that yeast activate β-oxidation and very long-chain fatty acid metabolism to reinforce membrane barrier function under stress [25,26].

#### 3.6.3 Methanol-specific transcriptional changes

Among the 994 DEGs specific to the methanol-stress comparison (GS115-28-5 vs. ATH-28-5), cell cycle and nuclear division pathways were significantly enriched (Fig. 7D–F; Tables S7 and 9), suggesting that ATH maintains proliferative capacity under methanol stress. Additionally, transmembrane transport-related genes were downregulated, which may reflect reduced uptake of toxic metabolites, complementing the enhanced formaldehyde clearance observed in Fig. 6D.

### 3.7 Genome sequencing analysis

We performed genomic resequencing on strain ATH and its original strain GS115. In the entire genome of the mutant strain ATH, a total of 19 variant sites were identified that differ from GS115. These variations included 9 SNPs and 10 InDels (Table S10). Subsequently, the genes harboring these variants were examined in the transcriptomic datasets obtained under high-temperature and high-methanol conditions, and four genes were shown to be downregulated under both conditions. These four genes are PAS_chr1-1_0203 (general amino acid permease), PAS_chr2-2_0367 (PDZ domain-containing signaling-associated protein), PAS_chr1-1_0097 (cyclin), and PAS_chr2-1_0870 (6-phosphofructo-2-kinase) (Table S11).

## 4 Discussion

In this study, we developed a mutant strain, ATH, using a combined ARTP mutagenesis and ALE strategy. Under individual stress conditions, ATH exhibited robust growth at 37 °C and tolerated 5% (v/v) methanol, achieving over 3-fold and 8-fold higher cell densities than the parental GS115, respectively. Previous efforts to improve *K. phaffii* robustness have largely focused on single-stress tolerance: Lin et al. isolated the thermotolerant strain G14 through ALE at 37 °C [7], while Wang et al. evolved the methanol-tolerant strain MMC-SFC, which withstood 10% methanol in BMMY [5]. However, these strains were optimized for only one type of stress. In contrast, ATH concomitantly acquired tolerance to both high temperature and high methanol, representing a rare case of cross-tolerance in methylotrophic yeasts. As an expression host, ATH further improved heterologous GFP production by more than 40% at 33 °C (Fig. 3), demonstrating industrial potential for reducing cooling costs while maintaining induction efficiency. To systematically elucidate the biological basis of this cross-tolerance, we conducted a comprehensive analysis across physiological and molecular levels. At the physiological level, ATH displayed enhanced cell wall/membrane integrity, reduced oxidative stress, and accelerated formaldehyde clearance under both stresses (Fig. 5, Fig. 6). Transcriptomic analysis further revealed that these improvements were underpinned by a shared transcriptional program involving coordinated upregulation of HSPs, antioxidant enzymes, and cell wall remodeling genes (Fig. 7), rather than isolated genetic changes.

Heat shock [27], methanol stress [28], and oxidative stress [29] are well-documented inducers of HSP families, which prevent protein misfolding and aggregation, promote refolding or targeted degradation, and ultimately maintain protein homeostasis. Consistent with this, ATH showed coordinated upregulation of the entire HSP system under both thermal and methanol stresses, including members of the HSP70, HSP40, and HSP90 families. Notably, Hsp104 and Hsp70-SSA2 exhibited the highest fold-changes (>3-fold), consistent with recent findings that HSPs serve as critical protectants for methylotrophic yeasts under stress conditions [30]. In *Pichia kudriavzevii* RZ8-1, heat shock at 42 °C induced marked upregulation of SSQ1 and HSP90, effectively reducing protein aggregation and restoring secretory capacity [31], while heterologous expression of OsHSP20 enhanced heat and salt tolerance in both *E. coli* and *K. phaffii* [32]. Moreover, as shown in *Saccharomyces cerevisiae*, the Skn7 response regulator interacts with Hsf1 in vivo and is required for induction of heat shock genes by oxidative stress [29], suggesting that the ROS reduction observed in ATH (Fig. 6B) may reinforce HSP expression through stress-signaling cross-talk. However, the full cross-tolerant phenotype likely requires synergistic action of the entire HSP network alongside antioxidant and cell wall defense pathways, rather than any single chaperone alone. When subjected to the combination of 37 °C and 5% methanol, ATH exhibited severely inhibited growth (Fig. S2), indicating that even a fully induced HSP network has limited buffering capacity when cellular stress exceeds a threshold.

Beyond protein homeostasis, enhanced cellular tolerance was closely associated with structural adaptations of the cell wall and membrane. Previous studies on stress-tolerant yeasts support this notion: Wang et al. reported that decreased membrane fluidity in MMC-SFC reduced formaldehyde accumulation by 75% [5], while Li et al. demonstrated that OLE1 overexpression enhances thermotolerance by increasing fatty acid unsaturation to reduce lipid peroxidation [33]. Our physiological assays revealed that ATH possesses superior cell wall integrity under CR and SDS challenge. In addition, ATH displayed reduced PI permeability and elevated membrane ATPase activity, indicative of enhanced membrane integrity (Fig. 5). These phenotypes were mirrored at the transcriptional level by upregulation of β-1,3-glucan transferases, exo-glucanases, and chitin transglycosidases (Fig. 7; Table S3), indicating active cell wall remodeling. In the context of methanol stress, Kim et al. recently identified mutations in membrane-associated phosphatases (PSR1) as evolutionary targets in methanol-tolerant strains, further underscoring the conserved role of membrane integrity across independently evolved lineage [34]**.**

High temperature [7], methanol stress [35], and heterologous protein expression [8] can all lead to intracellular ROS accumulation, triggering lipid peroxidation and protein damage [36]. Our results showed that ATH maintains significantly lower ROS and MDA levels under both stresses (Fig. 6B and C), together with upregulation of catalase and mitochondrial peroxiredoxin (Fig. 7). The antioxidant properties of ATH are consistent with established frameworks across diverse organisms: the heat-tolerant strain G14 enhances peroxisomal ROS buffering, improving cross-tolerance to heat and oxidative stress [7]; thermophilic fungi exhibit SOD and CAT enzymes with excellent thermal stability, enabling them to cope with oxidative stress at high temperatures [37]; and in *K. phaffii,* heterologous expression of CAT effectively reduces H_2_O_2_ levels [38]. Similarly, the methanol-tolerant mutant MMC-SFC maintains low oxidative stress during early fermentation, contributing to its methanol resistance [5], while Mejía-Barajas et al. documented enhanced antioxidant responses in a thermotolerant yeast [23]. Importantly, ATH also exhibited faster extracellular formaldehyde clearance (Fig. 6D), which may reflect enhanced metabolic detoxification, via upregulation of FLD, FGH, FDH, and DAS (Table S5), and possibly reduced formaldehyde influx associated with improved membrane integrity. Recent ALE work by Kim et al. similarly identified formaldehyde management as a key tolerance mechanism, with BFA1 mutations contributing to lower extracellular formaldehyde accumulation in evolved strains [34], while Cai et al. reported metabolic regulation adapting to high methanol environments in methylotrophic yeasts *Ogataea methanolica* [35].

While the mechanisms described above collectively account for ATH's robustness under individual stresses, a notable feature of its transcriptional program is the significant downregulation of amino acid biosynthesis and secondary metabolite synthesis under both stresses (Fig. 7E and F; Tables S8 and S9). This implies that the evolved strains may have adopted a more economical metabolic strategy that reduces biosynthetic demand, although further investigation is needed. Consequently, when 37 °C and 5% methanol are combined, the cumulative metabolic demand for protein folding, membrane remodeling, and formaldehyde detoxification probably exceeds the cellular buffering capacity, resulting in growth arrest. This phenomenon aligns with the stress synergy principle documented in *S. cerevisiae*, where combined heat and oxidative stress cause more severe growth defects than either stress alone [19]. Such trade-offs are characteristic of evolved stress-tolerant strains and highlight the physiological limits of cross-tolerance.

Whole-genome resequencing distinguished ATH from GS115 by only 19 variant sites (9 SNPs and 10 InDels), of which four variant-harbouring genes, a general amino acid permease, a cyclin, a PDZ domain-containing protein and a 6-phosphofructo-2-kinase, were downregulated under both stresses (Table S11). These stable genomic changes, rather than transient transcriptional fluctuations, may contribute to the alterations in amino acid and carbon metabolism observed in ATH. Reverse-genetic validation of these candidates will be needed to establish their causal roles in heat–methanol cross-tolerance.

Despite these advances, several limitations remain. The inability of ATH to grow under combined 37 °C and 5% methanol suggests that additional engineering, such as strengthening formaldehyde detoxification or ER-associated degradation, may be needed to push the tolerance boundary. Integrating multi-omics approaches will provide a more comprehensive understanding of how ARTP-ALE shapes the cellular machinery for robust bioprocessing.

## 5 Conclusion

In this study, we developed a robust *K. phaffii* strain ATH with cross-tolerance to high temperature (37 °C) and high methanol (5%) through integrated ARTP mutagenesis and ALE. Under each individual stress, ATH achieved over 3-fold (heat) and 8-fold (methanol) higher cell densities than the parental GS115, and outperformed GS115 in heterologous expression of three proteins (GFP, OPH, and ChiA) at the elevated induction temperature of 33 °C. Physiologically, ATH exhibited lower ROS and MDA levels, improved cell wall/membrane integrity, and enhanced formaldehyde clearance. Transcriptomic analysis revealed that a shared transcriptional program underlying this cross-tolerance, featuring coordinated upregulation of HSPs, antioxidant enzymes, and cell wall remodeling genes under both stresses, while whole-genome resequencing identified only 19 genetic variants, suggesting a compact mutational basis for the evolved phenotype. Together, this work provides an improved *K. phaffii* strain with enhanced stress resilience and protein production capacity, and elucidates the system-level mechanisms underlying cross-stress adaptation, offering insights into future engineering of robust industrial microorganisms. Nevertheless, ATH failed to grow under the simultaneous 37 °C and 5% methanol co-stress, indicating that further engineering, such as strengthening formaldehyde detoxification, will be required for industrial applications under combined stress.

## CRediT authorship contribution statement

**Chaoyin Li:** Writing – original draft, Visualization, Software, Methodology, Investigation, Formal analysis. **Tianzhi Jiang:** Methodology, Data curation. **Yesong Bo:** Validation, Software, Methodology. **Zhenghui Lu:** Validation, Methodology, Investigation, Funding acquisition. **Yuling Zhou:** Writing – review & editing, Writing – original draft, Supervision. **Guimin Zhang:** Writing – review & editing, Writing – original draft, Supervision, Funding acquisition, Conceptualization.

## Data availability statement

Data available on request from the authors.

## Ethics approval statement

The study in this article did not involve any trials on humans or animals.

## Funding statement

This work was supported by 10.13039/501100012166National Key R&D Program of China (2022YFA0911800), 10.13039/501100004826Beijing Natural Science Foundation (L244001) and Project “Vice President of Science and Technology” of Changping District, Beijing (202501003003).

## Declaration of competing interest

The authors declare the following financial interests/personal relationships which may be considered as potential competing interests:

This work was supported by the National Key R&D Program of China (Grant No. 2022YFA0911800), the Beijing Natural Science Foundation (Grant No. L244001), and the Project "Vice President of Science and Technology" of Changping District, Beijing (Grant No. 202501003003). The authors have no other financial interests or personal relationships that could have influenced the work reported in this paper.
