# Supplementary material for: Screening a thermotolerant Komagataella phaffii strain with methanol cross-tolerance and the associated physiological and transcriptional responses
Source: Synth Syst Biotechnol. 2026 Oct 15;19:57–68. doi: 10.1016/j.synbio.2026.10.003 (PMC13693071; doi:10.1016/j.synbio.2026.10.003)
Supplement: Supplementary file 1 [file mmc1.docx]

**Table S1.** Summary of RNA-Seq reads obtained in this study.

|  | Total Raw Reads | Total Clean Reads | Mapped Rate (%) | Unique Mapped Rate (%) | Q20 (%) | Q30 (%) |
| --- | --- | --- | --- | --- | --- | --- |
| ATH-28-1-1 | 50368392 | 45861446 | 96.86 | 96.84 | 98.11 | 94.03 |
| ATH-28-1-2 | 39535474 | 37091260 | 97.32 | 97.31 | 98.66 | 95.8 |
| ATH-28-1-3 | 38571376 | 35484768 | 97.8 | 97.79 | 98.22 | 94.38 |
| ATH-37-1-1 | 45388954 | 41600332 | 97.4 | 97.31 | 98.23 | 94.4 |
| ATH-37-1-2 | 52783346 | 47962658 | 97.06 | 96.99 | 98.11 | 94 |
| ATH-37-1-3 | 44977736 | 40295372 | 97.07 | 96.98 | 98.03 | 93.77 |
| ATH-28-5-1 | 39141796 | 36624598 | 98.21 | 98.1 | 98.46 | 95.16 |
| ATH-28-5-2 | 46743152 | 42574370 | 98.19 | 98.07 | 98.1 | 94.03 |
| ATH-28-5-3 | 49238384 | 45006656 | 98.06 | 97.94 | 98.15 | 94.17 |
| GS115-28-1-1 | 39440056 | 36411634 | 97.97 | 97.85 | 98.27 | 94.52 |
| GS115-28-1-2 | 51686776 | 47877442 | 98.14 | 98 | 98.36 | 94.79 |
| GS115-28-1-3 | 53268730 | 49415570 | 98.12 | 97.98 | 98.35 | 94.83 |
| GS115-37-1-1 | 44394628 | 40121112 | 97.82 | 97.77 | 97.99 | 93.6 |
| GS115-37-1-2 | 48874096 | 44022944 | 97.65 | 97.6 | 98.02 | 93.71 |
| GS115-37-1-3 | 44590824 | 40710070 | 97.69 | 97.64 | 98.15 | 94.17 |
| GS115-28-5-1 | 39440056 | 36482200 | 97.45 | 97.4 | 98.38 | 94.89 |
| GS115-28-5-2 | 40320082 | 38862924 | 97.86 | 97.81 | 99.2 | 97.56 |
| GS115-28-5-3 | 39382692 | 36199826 | 97.35 | 97.3 | 98.22 | 94.35 |

**Table S2.** Upregulated heat shock proteins in the GS115-37-1 vs. ATH-37-1 and GS115-28-5 vs. ATH-28-5 comparison groups.

| name | Log2 Fold Change  (GS115-37-1 vs ATH-37-1) | Log2 Fold Change  (GS115-28-5 vs ATH-28-5) | Gene description |
| --- | --- | --- | --- |
| PAS_chr3_0230 | 5.341 | 1.429 | ATPase involved in protein folding and the response to stress |
| PAS_chr1-3_0102 | 4.196 | 1.685 | Heat shock protein that cooperates with Ydj1p (Hsp40) and Ssa1p (Hsp70) |
| PAS_chr4_0552 | 4.044 | 1.774 | ATPase involved in protein folding and nuclear localization signal (NLS)-directed nuclear transport |
| PAS_chr3_0170 | 4.032 | 1.215 | Co-chaperone that binds to Hsp82p and activates its ATPase activity |
| PAS_chr2-1_0518 | 2.793 | 1.246 | Heat shock protein STI1 |
| PAS_chr2-2_0151 | 2.634 | 1.568 | Type II HSP40 co-chaperone that interacts with the HSP70 protein Ssa1p |
| PAS_chr3_0567 | 2.046 | 1.274 | Peptidyl-prolyl cis-trans isomerase (cyclophilin) |
| PAS_chr2-1_0140 | 1.952 | 1.337 | ATPase |
| PAS_chr2-1_0042 | 1.855 | 1.089 | Hsp70 (Ssa1p) nucleotide exchange factor, cytosolic homolog of Sil1p |
| PAS_chr2-1_0828 | 1.305 | 1.640 | Hsp90 co-chaperone Cdc37 |
| PAS_chr4_0353 | 1.204 | 1.960 | Specialized J-protein that functions with Hsp70 in Fe-S cluster biogenesis in mitochondria |

**Table S3.** Enriched genes in side of membrane (GO:0098552) and fungal-type cell wall (GO:0009277) in the GS115-37-1 vs. ATH-37-1 and GS115-28-5 vs. ATH-28-5 comparison groups.

| GO terms | name | Log2 Fold Change  (GS115-37-1 vs ATH-37-1) | Log2 Fold Change  (GS115-28-5 vs ATH-28-5) | Gene description |
| --- | --- | --- | --- | --- |
| side of membrane | PAS_chr4_0151 | 5.765 | 5.820 | Hemoglobin and hemoglobin-haptoglobin-binding protein |
|  | PAS_chr4_0559 | 3.254 | 2.864 | Putative chitin transglycosidase |
|  | PAS_chr1-3_0226 | 2.639 | 3.383 | Beta-1,3-glucanosyltransferase |
|  | PAS_chr2-1_0172 | 2.311 | 3.038 | Subtilase family |
|  | PAS_chr2-2_0148 | 2.290 | 2.570 | Cell wall protein that contains a putative GPI-attachment site |
|  | PAS_chr1-4_0586 | 2.169 | 2.264 | mmc protein |
|  | PAS_chr2-1_0454 | 2.155 | 2.679 | Major exo-1,3-beta-glucanase of the cell wall |
|  | PAS_chr4_0972 | 2.144 | 3.023 | Seripauperin and TIP1 family |
|  | PAS_chr1-3_0227 | 1.836 | 2.645 | Beta-1,3-glucanosyltransferase |
|  | PAS_chr3_0076 | 1.565 | 6.915 | Cysteine-rich secretory protein family |
|  | PAS_chr1-1_0147 | 1.329 | 1.430 | Putative mannosidase, essential glycosylphosphatidylinositol (GPI)-anchored membrane protein |
|  | PAS_chr1-3_0229 | 1.262 | 3.113 | Glycosyl hydrolases family 17 |
|  | PAS_chr4_0113 | 1.180 | 1.491 | Peptidase family M1 domain |
|  | PAS_chr3_0303 | 1.066 | 1.606 | Aspartic protease, attached to the plasma membrane via a glycosylphosphatidylinositol (GPI) anchor |
|  | PAS_chr3_0015 | -1.106 | 3.882 | Flocculation protein FLO1 |
|  | PAS_chr2-1_0667 | -1.191 | -1.482 | Protein involved in negative regulation of transcription of iron regulon |
|  | PAS_chr3_0299 | -1.322 | -1.602 | Aspartic protease, attached to the plasma membrane via a glycosylphosphatidylinositol (GPI) anchor |
|  | PAS_chr3_0394 | -1.407 | -1.168 | Putative GPI-anchored aspartic protease |
|  | PAS_chr3_1157 | -1.507 | -1.710 | Belongs to the peptidase A1 family |
|  | PAS_FragB_0067 | -2.052 | -2.367 | Mucin-like protein |
|  | PAS_chr4_0493 | -3.040 | -2.520 | Histidine phosphatase superfamily (branch 1) |
| fungal-type cell wall | PAS_chr4_0151 | 5.765 | 5.820 | Hemoglobin and hemoglobin-haptoglobin-binding protein |
|  | PAS_chr4_0305 | 5.266 | 5.980 | O-glycosylated protein required for cell wall stability |
|  | PAS_chr2-1_0887 | 3.290 | 3.200 | Cell wall mannoprotein PIR3 |
|  | PAS_chr4_0559 | 3.254 | 2.864 | Putative chitin transglycosidase |
|  | PAS_chr1-3_0226 | 2.639 | 3.383 | Beta-1,3-glucanosyltransferase |
|  | PAS_chr2-1_0355 | 2.518 | 3.710 | WD domain, G-beta repeat |
|  | PAS_chr2-2_0148 | 2.290 | 2.570 | Cell wall protein that contains a putative GPI-attachment site |
|  | PAS_chr1-4_0586 | 2.169 | 2.264 | mmc protein |
|  | PAS_chr2-1_0454 | 2.155 | 2.679 | Major exo-1,3-beta-glucanase of the cell wall |
|  | PAS_chr4_0972 | 2.144 | 3.023 | Seripauperin and TIP1 family |
|  | PAS_chr4_0287 | 2.005 | 2.654 | Dicarboxylic amino acid permease |
|  | PAS_chr1-3_0227 | 1.836 | 2.645 | Beta-1,3-glucanosyltransferase |
|  | PAS_chr4_0307 | 1.615 | 1.303 | Beta-glucosidase |
|  | PAS_chr3_0076 | 1.565 | -1.861 | Cysteine-rich secretory protein family |
|  | PAS_chr1-1_0147 | 1.329 | 6.915 | Putative mannosidase, essential glycosylphosphatidylinositol (GPI)-anchored membrane protein |
|  | PAS_chr2-1_0052 | 1.297 | 1.430 | RAM signalling pathway protein |
|  | PAS_chr1-3_0229 | 1.262 | 3.556 | Glycosyl hydrolases family 17 |
|  | PAS_chr4_0113 | 1.180 | 3.113 | Peptidase family M1 domain |
|  | PAS_chr3_0303 | 1.066 | 1.491 | Aspartic protease, attached to the plasma membrane via a glycosylphosphatidylinositol (GPI) anchor |
|  | PAS_chr2-1_0129 | -1.025 | 1.606 | Proline permease |
|  | PAS_chr3_0015 | -1.106 | -1.541 | Flocculation protein FLO1 |
|  | PAS_chr3_0299 | -1.322 | 3.882 | Aspartic protease, attached to the plasma membrane via a glycosylphosphatidylinositol (GPI) anchor |
|  | PAS_chr2-1_0659 | -1.341 | -1.602 | Proline permease, required for high-affinity transport of proline |
|  | PAS_chr3_0394 | -1.407 | -2.127 | Putative GPI-anchored aspartic protease |
|  | PAS_chr3_1157 | -1.507 | -1.168 | Belongs to the peptidase A1 family |
|  | PAS_chr1-1_0203 | -1.736 | -1.710 | General amino acid permease |

**Table S4.** Enriched genes in cellular oxidant detoxification (GO:0098869) in the GS115-37-1 vs. ATH-37-1 and GS115-28-5 vs. ATH-28-5 comparison groups.

| name | Log2 Fold Change  (GS115-37-1 vs ATH-37-1) | Log2 Fold Change  (GS115-28-5 vs ATH-28-5) | Gene description |
| --- | --- | --- | --- |
| PAS_chr2-2_0131 | 1.795 | 1.303 | Catalase A |
| PAS_chr1-3_0160 | 1.327 | 1.234 | Protein adenylyltransferase SelO |
| PAS_chr3_0906 | 1.054 | 1.436 | Mitochondrial peroxiredoxin (1-Cys Prx) with thioredoxin peroxidase activity |
| PAS_chr3_0355 | -3.192 | -3.168 | Carboxymuconolactone decarboxylase family |
| PAS_chr2-1_0315 | -1.856 | -1.151 | Omega class glutathione transferase |
| PAS_chr2-2_0382 | -1.133 | -1.202 | Thiol peroxidase that functions as a hydroperoxide receptor |

**Table S5.** Differential expression of formaldehyde clearance‑related genes in the ATH strain under high‑temperature (GS115-37-1 vs. ATH-37-1) and high‑methanol (GS115-28-5 vs. ATH-28-5) stress conditions.

| name | regulated | |
| --- | --- | --- |
|  | GS115-37-1 vs ATH-37-1 | GS115-28-5 vs ATH-28-5 |
| AOX | up | up |
| FLD | up | NS |
| FGH | up | NS |
| FDH | up | NS |
| DAS | up | NS |
| GSH1/GSH2 | down | up |
| CAT | up | up |

**Table S6.** GO terms for GS115-37-1 vs. ATH-37-1 comparison.

| **ONTOLOGY** | **ID** | **Description** | **Count** | **Up Count** | **Down Count** |
| --- | --- | --- | --- | --- | --- |
| CC | GO:0005782 | peroxisomal matrix | 40 | 30 | 10 |
| CC | GO:0005777 | peroxisome | 78 | 52 | 26 |
| MF | GO:0016491 | oxidoreductase activity | 67 | 33 | 34 |
| BP | GO:0006635 | fatty acid beta-oxidation | 27 | 23 | 4 |
| BP | GO:0009820 | alkaloid metabolic process | 22 | 9 | 13 |
| CC | GO:0009277 | fungal-type cell wall | 39 | 30 | 9 |
| CC | GO:0005759 | mitochondrial matrix | 100 | 56 | 44 |
| MF | GO:0030170 | pyridoxal phosphate binding | 23 | 3 | 20 |
| MF | GO:0005507 | copper ion binding | 39 | 24 | 15 |
| BP | GO:0000038 | very long-chain fatty acid metabolic process | 11 | 2 | 9 |
| BP | GO:0008652 | amino acid biosynthetic process | 15 | 1 | 14 |
| BP | GO:0008615 | pyridoxine biosynthetic process | 9 | 4 | 5 |
| BP | GO:0140723 | patulin biosynthetic process | 11 | 7 | 4 |
| CC | GO:0048046 | apoplast | 35 | 18 | 17 |
| CC | GO:0098552 | side of membrane | 24 | 15 | 9 |
| BP | GO:0033540 | fatty acid beta-oxidation using acyl-CoA oxidase | 8 | 8 | - |
| BP | GO:0009113 | purine nucleobase biosynthetic process | 8 | - | 8 |
| MF | GO:0051287 | NAD binding | 26 | 12 | 14 |
| MF | GO:2001070 | starch binding | 7 | 4 | 3 |
| CC | GO:0005778 | peroxisomal membrane | 41 | 35 | 6 |

**Table S7**. GO terms for GS115-28-5 vs. ATH-28-5 comparison

| **ONTOLOGY** | **ID** | **Description** | **Count** | **Up Count** | **Down Count** |
| --- | --- | --- | --- | --- | --- |
| CC | GO:0032126 | eisosome | 22 | 11 | 11 |
| CC | GO:0009277 | fungal-type cell wall | 56 | 42 | 14 |
| MF | GO:0016597 | amino acid binding | 29 | 6 | 23 |
| MF | GO:0015192 | L-phenylalanine transmembrane transporter activity | 14 | 4 | 10 |
| MF | GO:0015293 | symporter activity | 43 | 13 | 30 |
| BP | GO:0000280 | nuclear division | 16 | 12 | 4 |
| BP | GO:0008652 | amino acid biosynthetic process | 20 | 3 | 17 |
| CC | GO:0071944 | cell periphery | 147 | 78 | 69 |
| BP | GO:0031505 | fungal-type cell wall organization | 84 | 54 | 30 |
| BP | GO:0015801 | aromatic amino acid transport | 14 | 4 | 10 |
| BP | GO:0044406 | adhesion of symbiont to host | 24 | 15 | 9 |
| BP | GO:0009098 | L-leucine biosynthetic process | 16 | 2 | 14 |
| BP | GO:0006525 | arginine metabolic process | 18 | 5 | 13 |
| BP | GO:0015805 | S-adenosyl-L-methionine transport | 19 | 4 | 15 |
| MF | GO:0000095 | S-adenosyl-L-methionine transmembrane transporter activity | 19 | 4 | 15 |
| CC | GO:1903561 | extracellular vesicle | 56 | 32 | 24 |
| CC | GO:0098552 | side of membrane | 33 | 22 | 11 |
| MF | GO:0022857 | transmembrane transporter activity | 74 | 19 | 55 |
| CC | GO:0000324 | fungal-type vacuole | 143 | 68 | 75 |
| BP | GO:0015848 | spermidine transport | 19 | 6 | 13 |

**Table S8**. KEGG pathways for GS115-37-1 vs. ATH-37-1 comparison.

| **ID** | **Description** | **Count** | **Up Count** | **Down Count** |
| --- | --- | --- | --- | --- |
| ko01100 | Metabolic pathways | 178 | 79 | 99 |
| ko01230 | Biosynthesis of amino acids | 51 | 6 | 45 |
| ko01110 | Biosynthesis of secondary metabolites | 80 | 26 | 54 |
| ko04146 | Peroxisome | 20 | 18 | 2 |
| ko01210 | 2-Oxocarboxylic acid metabolism | 17 | 1 | 16 |
| ko00680 | Methane metabolism | 14 | 8 | 6 |
| ko00410 | beta-Alanine metabolism | 11 | 7 | 4 |
| ko00360 | Phenylalanine metabolism | 9 | 5 | 4 |
| ko01200 | Carbon metabolism | 31 | 20 | 11 |
| ko00300 | Lysine biosynthesis | 8 | - | 8 |
| ko00071 | Fatty acid degradation | 8 | 7 | 1 |
| ko00290 | Valine, leucine and isoleucine biosynthesis | 7 | - | 7 |
| ko00260 | Glycine, serine and threonine metabolism | 14 | 2 | 12 |
| ko00400 | Phenylalanine, tyrosine and tryptophan biosynthesis | 10 | 1 | 9 |
| ko00340 | Histidine metabolism | 8 | 1 | 7 |
| ko00750 | Vitamin B6 metabolism | 6 | - | 6 |
| ko00350 | Tyrosine metabolism | 7 | 4 | 3 |
| ko00380 | Tryptophan metabolism | 8 | 3 | 5 |
| ko00220 | Arginine biosynthesis | 8 | 2 | 6 |
| ko00250 | Alanine, aspartate and glutamate metabolism | 11 | 1 | 10 |

**Table S9**. KEGG pathways for GS115-28-5 vs. ATH-28-5 comparison.

| **ID** | **Description** | **Count** | **Up Count** | **Down Count** |
| --- | --- | --- | --- | --- |
| ko01230 | Biosynthesis of amino acids | 62 | 6 | 56 |
| ko01110 | Biosynthesis of secondary metabolites | 116 | 39 | 77 |
| ko01100 | Metabolic pathways | 245 | 107 | 138 |
| ko00250 | Alanine, aspartate and glutamate metabolism | 21 | 5 | 16 |
| ko01210 | 2-Oxocarboxylic acid metabolism | 22 | 2 | 20 |
| ko00220 | Arginine biosynthesis | 12 | 1 | 11 |
| ko00400 | Phenylalanine, tyrosine and tryptophan biosynthesis | 13 | - | 13 |
| ko04111 | Cell cycle - yeast | 42 | 35 | 7 |
| ko00290 | Valine, leucine and isoleucine biosynthesis | 8 | - | 8 |
| ko00300 | Lysine biosynthesis | 9 | - | 9 |
| ko04113 | Meiosis - yeast | 31 | 23 | 8 |
| ko00910 | Nitrogen metabolism | 5 | - | 5 |
| ko00650 | Butanoate metabolism | 6 | 4 | 2 |
| ko04392 | Hippo signaling pathway - multiple species | 6 | 6 | - |
| ko00260 | Glycine, serine and threonine metabolism | 16 | 3 | 13 |
| ko00630 | Glyoxylate and dicarboxylate metabolism | 12 | 4 | 8 |
| ko00330 | Arginine and proline metabolism | 11 | 1 | 10 |
| ko00750 | Vitamin B6 metabolism | 6 | - | 6 |
| ko00660 | C5-Branched dibasic acid metabolism | 3 | - | 3 |
| ko00340 | Histidine metabolism | 8 | - | 8 |

**Table S10**. Results from the whole genome sequence analysis. pos, position where the mutation is found; ref, reference sequence; alt, alternate sequence.

| **Region** | **Gene** | **Chrom** | **Pos** | **Ref** | **Alt** | **Mutation**  **_Type** |
| --- | --- | --- | --- | --- | --- | --- |
| **SNP** | | | | | | |
| Exonic | gene-PAS_chr3_0893 | NC_012965.1 | 1737527 | G | A | nonsynonymous SNV |
| Exonic | gene-PAS_chr1-1_0203 | NC_012963.1 | 952011 | A | G | nonsynonymous  SNV |
| Exonic | gene-PAS_chr1-1_0318 | NC_012963.1 | 1159986 | C | T | synonymous SNV |
| Upstream; Downstream | gene-PAS_chr1-4_0664(dist=58); gene-PAS_chr1-4_0263(dist=225) | NC_012963.1 | 1881387 | A | T |  |
| Upstream | gene-PAS_chr2-1_0593, gene-PAS_chr2-1_0594(dist=31) | NC_012964.1 | 1106135 | T | A |  |
| Upstream | gene-PAS_chr2-1_0784, gene-PAS_chr2-1_0785(dist=749) | NC_012964.1 | 1499297 | G | T |  |
| Upstream; Downstream | gene-PAS_chr2-2_0433, gene-PAS_chr2-2_0434(dist=62); gene-PAS_chr2-2_0435(dist=758) | NC_012964.1 | 1574562 | G | C |  |
| Upstream; Downstream | gene-PAS_chr2-2_0368(dist=167); gene-PAS_chr2-2_0367, gene-PAS_chr2-2_0369, gene-PAS_chr2-2_0370(dist=167) | NC_012964.1 | 1696936 | C | T |  |
| Downstream | gene-PAS_chr4_0432, gene-PAS_chr4_0433(dist=530) | NC_012966.1 | 852688 | G | A |  |
| **Indel** | | | | | |  |
| Frameshift  Insertion | gene-PAS_chr2-2_0402 | NC_012964.1 | 1642706 | C | CA |  |
| Upstream | gene-PAS_chr1-3_0011(dist=373) | NC_012963.1 | 21316 | C | CA |  |
| Upstream; Downstream | gene-PAS_chr1-1_0097(dist=320); gene-PAS_chr1-1_0096(dist=43) | NC_012963.1 | 736040 | A | AT |  |
| Upstream | gene-PAS_chr2-1_0237, gene-PAS_chr2-1_0238(dist=440) | NC_012964.1 | 446222 | C | CA |  |
| Upstream; Downstream | gene-PAS_chr2-1_0459(dist=204); gene-PAS_chr2-1_0460(dist=61) | NC_012964.1 | 848792 | C | CT |  |
| Upstream; Downstream | gene-PAS_chr2-1_0870(dist=85); gene-PAS_chr2-1_0624(dist=291) | NC_012964.1 | 1168792 | G | GA |  |
| Upstream | gene-PAS_chr2-2_0003(dist=379) | NC_012964.1 | 2390655 | A | AT |  |
| Upstream; Downstream | gene-PAS_chr3_0417(dist=514); gene-PAS_chr3_0418(dist=286) | NC_012965.1 | 813381 | A | AT |  |
| Upstream; Downstream | gene-PAS_chr4_0857(dist=283); gene-PAS_chr4_0858(dist=160) | NC_012966.1 | 1611406 | C | CTT |  |
| Downstream | gene-PAS_chr2-2_0010, gene-PAS_chr2-2_0011(dist=819) | NC_012964.1 | 2375713 | C | CT |  |

**Table S11**. Genomic and transcriptomic alterations of the four candidate genes in strain ATH.

| **name** | **Description** | **genome mutation** | **regulated** | |
| --- | --- | --- | --- | --- |
|  |  |  | **GS115-37-1 vs ATH-37-1** | **GS115-28-5 vs ATH-28-5** |
| PAS_chr1-1_0203 | General amino acid permease | SNP | down | down |
| PAS_chr2-2_0367 | PDZ domain-containing signaling-associated protein | SNP | down | down |
| PAS_chr1-1_0097 | Cyclin | INDEL | down | down |
| PAS_chr2-1_0870 | 6-phosphofructo-2-kinase/fructose-2,6-bisphosphatase | INDEL | down | down |

**Fig. S1**. Growth curves of GS115 and ATH at 28 °C in BMMY medium supplemented with 1% and 5% (v/v) methanol every 24 h. Data are presented as mean ± SD (n = 3 biological replicates).

**Fig. S2**. Growth curves of GS115 and ATH at 37 °C in BMMY medium supplemented with 5% (v/v) methanol. Data are presented as mean ± SD (n = 3 biological replicates).

**Fig. S3**. GFP expression in GS115 and ATH strains at 37 °C for 72 h. GS115-GFP and ATH-GFP were fermented in BMMY medium with 1% (v/v) methanol added every 12 hours. Fluorescence intensity normalized to OD_600_ was determined. Data are presented as mean ± SD (n = 3 biological replicates).
